# Comparative residue depletion and tissue distribution of three β-adrenergic agonists in broiler chickens

**DOI:** 10.3389/fvets.2026.1967907

**Published:** 2026-09-23

**Authors:** Defrim Veseli, Stefan Jovanov, Zehra Hajrulai-Musliu

**Affiliations:** Department of Chemistry, Faculty of Veterinary Medicine, Ss. Cyril and Methodius University in Skopje, Skopje, North Macedonia

**Keywords:** broiler chickens, food safety, LC–MS/MS, residue depletion, β-Adrenergic agonists

## Abstract

β-Adrenergic agonists are banned as growth promoters in the European Union, and clenbuterol and salbutamol are routinely screened in poultry under national residue-control programs; characterizing their residue behavior in edible tissues therefore supports the detection of illicit use. This study examined the residue depletion and tissue distribution of salbutamol, clenbuterol, and terbutaline in 168 Ross 308 broiler chickens. Each compound was administered in drinking water at 2.5 or 5 mg/L for seven consecutive days, chosen to model a covert exposure route rather than an authorized feed protocol, followed by a withdrawal period. Concentrations in breast muscle, liver, and kidney were quantified using a validated LC–MS/MS method compliant with Commission Implementing Regulation (EU) 2021/808. Residue values are group mean concentrations of three birds per group per sampling point (*n* = 3); because the individual per-bird results were not retained, no measures of dispersion are reported for the residue data. Quantifiable residues were detected in all three tissues at the first post-treatment sampling point, with the highest concentrations in kidney and liver and the lowest in breast muscle. Residues declined over the withdrawal period; terbutaline depleted most rapidly and clenbuterol persisted longest, and apparent depletion half-lives derived by log-linear regression were 1.1–1.5 days in liver and 0.7–1.2 days in kidney. By Day 46, all residues were below their respective matrix-specific decision limits (CCα), and no growth-promoting effect was observed for any compound at either concentration. Under this short drinking-water exposure, the three β-agonists showed distinct, tissue-dependent residue-depletion profiles but no measurable growth-promoting response in broilers. The comparative dataset, referenced to validated CCα values, supports residue surveillance and food-safety risk assessment of β-agonist residues in poultry.

## 1 Introduction

β-Adrenergic agonists are synthetic sympathomimetic compounds that interact with β-adrenergic receptors distributed across numerous tissues, including skeletal muscle, adipose tissue, the cardiovascular system, and the respiratory tract. These receptors are classified into β₁, β₂, and β₃ subtypes, each mediating distinct physiological responses. β₁-Receptors primarily regulate cardiac function, whereas β₂-receptors are involved in smooth-muscle relaxation, bronchodilation, and modulation of metabolic processes. β₃-Receptors play important roles in lipid mobilization and energy metabolism within adipose tissue (1–3). The biological effects of β-agonists depend on receptor selectivity, dose, duration of exposure, and species-specific physiological characteristics (4, 5). Clenbuterol, salbutamol, and terbutaline are selective β₂-adrenergic agonists that have been extensively investigated in food-producing animals.

In mammalian livestock species, β₂-agonists have been reported to promote lean tissue deposition and improve carcass composition through alterations in protein and lipid metabolism (1). Such nutrient-repartitioning effects have been documented in cattle, pigs, and sheep (6–10). Consequently, several β-agonists have a long history of legal or illegal use as growth-promoting agents in food production systems. In poultry, however, the evidence is less consistent. Although some studies have reported improvements in feed efficiency, carcass composition, or muscle deposition following administration of β-agonists, these effects are influenced by factors such as compound selection, dose, duration of exposure, diet composition, age, and sex (1, 6, 11–13). Other investigations have reported little or no enhancement of growth performance. The variable response observed in poultry has been attributed to differences in adrenergic receptor distribution and regulation relative to mammalian species. Broiler skeletal muscle appears to possess a lower density of functional β₂-adrenergic receptors, and downstream intracellular signaling mechanisms may be less responsive than those reported in mammals (1, 11). As a result, the growth-promoting efficacy of β-agonists in broilers remains uncertain and appears considerably less predictable than in conventional mammalian livestock species. Beyond questions of efficacy, β-agonists are of considerable regulatory importance because they may occur as residues in edible animal tissues. Consumption of foods containing β-agonist residues has been associated with adverse cardiovascular and neurological effects in humans, and numerous outbreaks of food poisoning linked to contaminated animal products have been documented (14–19). Clenbuterol, in particular, has frequently been associated with cases of illicit administration for growth promotion (20, 21). Consequently, strict regulatory controls and residue-monitoring programs have been established in many jurisdictions, including the European Union, where the use of β-agonists for growth-promotion purposes is prohibited and surveillance for residues forms part of official control programs (22–25). Reliable detection of β-agonist residues requires highly sensitive and selective analytical methodologies. Liquid chromatography coupled with tandem mass spectrometry (LC–MS/MS) has become the method of choice for residue monitoring because of its excellent sensitivity, specificity, and suitability for confirmatory analysis in complex biological matrices (26–28). Previous investigations have demonstrated tissue-dependent residue persistence, with residues often remaining detectable for longer periods in liver and kidney than in skeletal muscle (29–31). Understanding such depletion patterns is important for residue surveillance, regulatory enforcement, and food-safety risk assessment. Because β-agonists are prohibited as growth promoters in the European Union and clenbuterol and salbutamol are routinely screened in poultry tissues under National Residue Control Plans, the present study was undertaken primarily to characterize the tissue distribution and residue depletion of salbutamol, clenbuterol, and terbutaline in broiler chickens, in order to support the detection of potential illicit administration during residue surveillance. Administration via drinking water at 2.5 and 5 mg/L for 7 days was chosen deliberately to model a plausible covert route of exposure rather than to reproduce an authorized feed-based growth-promotion protocol; accordingly, the study was not designed to elicit maximal anabolic effects, and growth performance was monitored only as a secondary check on whether this regimen produced measurable responses. Residue concentrations in breast muscle, liver, and kidney were determined using a validated LC–MS/MS method. By comparing three β-agonists under identical experimental conditions, this study provides comparative information on residue persistence in edible poultry tissues and contributes to the scientific basis for residue-monitoring programs and food-safety assessment of β-agonist exposure in poultry production. To our knowledge, comparative residue-depletion data for salbutamol, clenbuterol, and terbutaline generated under identical experimental conditions in broiler chickens remain limited; the present study therefore provides a direct comparison of the tissue distribution and depletion characteristics of the three compounds, generating information relevant to surveillance strategies and risk-based residue monitoring.

## 2 Materials and methods

### 2.1 Animals and experimental design

A total of 168 one-day-old Ross 308 broiler chicks were obtained from a commercial hatchery (Veze Shari Company, North Macedonia). Upon arrival, birds were individually identified, weighed, and randomly allocated to seven experimental groups in a completely randomized design (CRD). The study comprised six treatment groups and one untreated control group, with each group consisting of 24 birds housed in four replicate floor pens, each containing six birds. Randomization was performed using a computer-generated allocation procedure to ensure similar initial body weights among groups and to minimize selection bias. The experimental design evaluated three β-adrenergic agonists, salbutamol, clenbuterol, and terbutaline, each administered at two concentrations (2.5 and 5 mg/L) via drinking water, resulting in six treated groups in addition to the control. Administration through drinking water was selected as a practical means of providing group-level exposure and was intended to simulate a potential route by which β-agonists could be administered under field conditions. Because water intake was not measured individually, the exact dose received by each bird could not be determined; therefore, the data represent group-level rather than individual exposure. Birds were reared as a mixed-sex population, and sex was not included as an experimental factor. All groups were maintained under identical environmental and management conditions throughout the study. The β-agonists were administered for seven consecutive days (Days 13–19 of age), after which all birds received untreated drinking water and a basal diet until the end of the experiment (Day 46). This post-treatment period was designated the withdrawal phase and used to evaluate residue depletion in edible tissues.

Tissue sampling was performed at predetermined intervals during the withdrawal period (Days 21, 22, 23, 25, 28, 30, 39, and 46). At each sampling point, three birds per treatment group were randomly selected and euthanized for residue analysis. Selection was performed without replacement to avoid repeated sampling of the same animals and to minimize sampling bias. The study design therefore generated residue-depletion data from independent animals at each time point while allowing concurrent assessment of growth performance throughout the experimental period.

### 2.2 Housing and management

Birds were housed in floor pens bedded with fresh wood shavings and managed according to the Ross 308 Broiler Management Guidelines (32). Each pen contained six birds, and stocking density was maintained at approximately 10 birds per m^2^. For growth-performance analyzes, the pen was considered the experimental unit. Environmental conditions were standardized across all treatment groups throughout the study. Ambient temperature was maintained at 32 °C–33 °C during the first week of life and subsequently reduced by approximately 2 °C–3 °C per week until reaching 22 °C–24 °C by Day 21. Relative humidity was maintained between 50% and 60%, and ventilation was provided by an automated negative-pressure system designed to maintain acceptable air quality and ammonia concentrations below 10 ppm.

A lighting program consistent with Ross 308 recommendations was implemented, consisting of 23 h of light on Day 1, followed by a daily dark period from the second week onward and near-continuous illumination during the final 3 days before slaughter. Feed and water were provided ad libitum throughout the experiment. Birds received commercially formulated diets in three feeding phases: starter (Days 1–10; 22% crude protein, 3,000 kcal ME/kg), grower (Days 11–28; 20% crude protein, 3,100 kcal ME/kg), and finisher (Days 29–46; 18% crude protein, 3,200 kcal ME/kg). All diets were formulated to meet or exceed the nutritional requirements of Ross 308 broilers. Apart from the experimental β-adrenergic agonists administered to the treatment groups, no growth promoters, feed additives with anabolic activity, or other veterinary medicinal products were used during the study.

### 2.3 β-Agonist administration

Salbutamol, clenbuterol, and terbutaline (purity ≥ 98%; Sigma-Aldrich, St. Louis, MO, USA) were administered via drinking water at nominal concentrations of 2.5 and 5 mg/L. Stock solutions were prepared in distilled water and diluted daily to the required concentrations to ensure homogeneous distribution and maximize stability throughout the treatment period. Administration was conducted for seven consecutive days (Days 13–19 of age) under identical husbandry and management conditions for all treated groups. Medicated water was prepared fresh each day and supplied ad libitum. Following the treatment period, all birds received untreated drinking water and the basal diet until Day 46, thereby establishing a withdrawal phase to evaluate tissue residue depletion. Administration via drinking water was selected as a practical route to achieve group-level exposure and evaluate subsequent residue persistence under conditions relevant to potential field exposure. However, because individual water consumption was not measured, the exact dose each bird received could not be determined. Consequently, exposure should be regarded as group-based rather than individual-based, and variation in water intake among birds may have contributed to variability in tissue residue concentrations. Based on Ross 308 reference water-intake values (32) and the body weights recorded over the treatment window (Days 13–19), average water consumption was of the order of 0.10–0.15 L per bird per day at a mean body weight of approximately 0.6 kg. On this basis, the nominal drinking-water concentrations of 2.5 and 5 mg/L correspond to approximate group-level intakes of about 0.5 and 1.0 mg/kg body weight per day, respectively. These figures are indicative group-level approximations only, as individual water intake was not recorded.

### 2.4 Growth performance assessment

Body weight was measured at predefined time points (Days 20, 22, 24, 26, 33, and 40 of age) using a calibrated digital scale (Mettler Toledo PB3002-S, Switzerland; precision ± 1 g). For growth-performance analyzes, the pen was considered the experimental unit. To reduce variation associated with gastrointestinal fill, birds were fasted for approximately 8 h prior to each weighing. General health status and mortality were monitored daily in all groups during both the treatment and withdrawal phases. Birds were observed for clinical signs potentially associated with β-adrenergic agonist administration, including changes in behavior, activity, feed consumption, and overall condition. No treatment-related clinical abnormalities were observed during the study, and no mortality attributable to the experimental treatments occurred. These observations indicate that the β-agonist regimens administered were well tolerated under the experimental conditions.

### 2.5 Tissue sampling for residue analysis

Residue depletion was evaluated during the withdrawal period at predefined sampling times corresponding to Days 21, 22, 23, 25, 28, 30, 39, and 46 of age. At each sampling point, three birds were randomly selected from each experimental group and euthanized by cervical dislocation following a 12-h feed-withdrawal period, in accordance with the AVMA Guidelines for the Euthanasia of Animals (33). Random selection was performed without replacement to ensure that each bird contributed data only once. Immediately after euthanasia, tissue samples were collected for residue analysis, including breast muscle (pectoralis major, approximately 5 g), liver (left lobe, approximately 2 g), and kidney (pooled sample from both kidneys, approximately 2 g). To minimize post-mortem degradation, samples were transferred into pre-labeled sterile polypropylene containers, snap-frozen in liquid nitrogen immediately after collection, and stored at −20 °C until analysis. All samples were analyzed within 30 days of collection to preserve sample integrity and ensure the reliability of residue measurements. The selected tissues were chosen because they represent the principal edible tissues and organs commonly targeted in residue-monitoring programs for β-adrenergic agonists.

### 2.6 LC–MS/MS analysis

#### 2.6.1 Reagents and standards

Certified reference standards of clenbuterol, salbutamol, and terbutaline (purity ≥ 99%) and their corresponding stable-isotope-labeled internal standards (clenbuterol-d6, salbutamol-d9, and terbutaline-d9) were purchased from Sigma-Aldrich (St. Louis, MO, USA). HPLC-grade acetonitrile, methanol, and water were obtained from Merck KGaA (Darmstadt, Germany). Formic acid (≥98%) and ammonium formate were supplied by Sigma-Aldrich. Solid-phase extraction (SPE) clean-up was performed using Oasis MCX cartridges (60 mg/3 mL; Waters Corporation, Milford, MA, USA). All reagents were of analytical or LC–MS grade and were used without further purification.

#### 2.6.2 Sample preparation

Frozen tissue samples were thawed, homogenized using an IKA Ultra-Turrax T25 homogenizer (12000 rpm, 1 min), and diluted with phosphate-buffered saline (PBS; pH 7.4) at a ratio of 1:2 (w/v). Stable-isotope-labeled internal standards were added prior to extraction to compensate for matrix effects and analytical variability. Proteins were precipitated by the addition of acetonitrile (1:1, v/v), followed by centrifugation at 10,000 × *g* for 10 min at 4 °C. The resulting supernatant was subjected to SPE clean-up using Oasis MCX cartridges preconditioned with 3 mL methanol and 3 mL water. After sample loading, cartridges were washed with 5% aqueous methanol and analytes were eluted using 2% ammoniated methanol. The eluates were evaporated to dryness under a gentle stream of nitrogen at 40 °C and reconstituted in 200 μL of 0.1% formic acid in water prior to LC–MS/MS analysis.

#### 2.6.3 Chromatographic and mass-spectrometric conditions

Chromatographic separation was carried out using a Waters Acquity UPLC system coupled to a Waters Xevo TQ-S triple-quadrupole mass spectrometer (Waters Corporation, Milford, MA, USA). Analytes were separated on an Acquity UPLC BEH C18 column (2.1 × 100 mm, 1.7 μm) maintained at 40 °C.

The mobile phase consisted of (A) water containing 0.1% formic acid and 5 mM ammonium formate and (B) acetonitrile containing 0.1% formic acid. Gradient elution was programmed as follows: 0–1.0 min, 5% B; 1.0–4.0 min, linear increase from 5% to 95% B; 4.0–5.0 min, 95% B; 5.0–5.1 min, return to 5% B; and 5.1–7.0 min, re-equilibration at 5% B. The flow rate was 0.4 mL/min, the injection volume was 5 μL, and the total run time was 7 min. Mass-spectrometric detection was performed using electrospray ionization in positive-ion mode (ESI+). Instrument settings were as follows: capillary voltage 3.0 kV, source temperature 150 °C, desolvation temperature 400 °C, cone gas flow 100 L/h, and desolvation gas flow 800 L/h. Data acquisition was performed in multiple reaction monitoring (MRM) mode. Quantification was based on the designated quantifier transition (Q), while compound identity was confirmed using the corresponding qualifier transition (q). The monitored precursor and product ion transitions and associated instrument parameters are presented in Table 1. Data acquisition and processing were performed using MassLynx software version 4.1 (Waters Corporation).

**Table 1:** MRM transitions and instrument parameters for LC–MS/MS detection of the β-agonists.

Compound	Precursor ion (m/z)	Product ions (m/z)	Cone voltage (V)	Collision energy (eV)
Clenbuterol	277.0	203.0 (Q), 131.9 (q)	22	16, 30
Salbutamol	240.1	148.0 (Q), 166.0 (q)	22	20, 14
Terbutaline	226.1	152.0 (Q), 107.0 (q)	26	16
Clenbuterol-d6 (CLEN-D6)	283.03	203.56	16	22
Salbutamol-d9 (SALB-D9)	249.08	148.59	24	20
Terbutaline-d9 (TERB-D9)	235.07	152.83	16	34

Transitions correspond to those acquired in the specificity chromatograms (Supplementary Figure S1).

Q, quantifier transition; q, qualifier transition.

#### 2.6.4 Method validation

The analytical method was validated for breast muscle, liver, and kidney in accordance with Commission Implementing Regulation (EU) 2021/808 and was based on multi-residue LC–MS/MS procedures previously developed and validated in our laboratory (34–36). The principal validation parameters are summarized in Tables 2, 3. Linearity was evaluated using six matrix-matched calibration levels for each analyte–matrix combination. Calibration curves were generated by linear regression of analyte-to-internal-standard peak-area ratios against nominal concentrations. Method specificity was assessed by analyzing blank tissue samples from each matrix and confirming the absence of interfering signals at the target analytes’ retention times. Representative chromatograms obtained from blank and fortified samples are presented in Supplementary Figure S1. The decision limit (CCα) and detection capability (CCβ) were calculated in accordance with Commission Implementing Regulation (EU) 2021/808. Accuracy, recovery, repeatability (intra-day precision), and reproducibility (inter-day precision) were determined using blank tissue samples fortified at three concentration levels and analyzed in replicate. Precision was expressed as relative standard deviation (RSD). Residue concentrations are reported relative to the corresponding matrix-specific CCα values. Results below CCα are presented as “< CCα.” Because CCα represents a regulatory decision limit rather than a limit of detection or quantification, results reported as below CCα should be interpreted as non-confirmatory within the regulatory framework and not as evidence of the complete absence of residues. Method validation addressed the core performance characteristics required under Commission Implementing Regulation (EU) 2021/808, namely linearity, specificity, decision limit (CCα), detection capability (CCβ), accuracy, recovery, and precision. Three further groups of performance characteristics were not separately determined in the present validation, and are declared here as deviations from the full validation package specified in the Regulation. First, the relative matrix effect was not quantified independently. Quantification was performed against matrix-matched calibration curves with stable-isotope-labeled internal standards added to the test portion before extraction, which compensates for matrix-related signal suppression or enhancement, but this compensation is not a substitute for a formal determination of the matrix effect. Second, stability testing of the analytes in matrix and in standard solution, addressed in Section 2.5 of Annex I to the Regulation, was not carried out as part of this validation; freeze–thaw and processed-sample stability were likewise not evaluated. In mitigation, all samples were snap-frozen in liquid nitrogen immediately after collection, held at −20 °C, and analyzed within 30 days. Third, carry-over and dilution integrity, which represent good analytical practice rather than parameters specified in the Regulation, were not assessed. These deviations do not affect the confirmation of analyte identity, which was based on retention time together with one quantifier and one qualifier MRM transition, but they should be taken into account when the concentrations reported here are compared with results generated by fully validated official-control methods.

**Table 2:** Linearity, coefficient of determination (R²), decision limit (CCα), and detection capability (CCβ) for clenbuterol, salbutamol, and terbutaline in muscle, liver, and kidney.

Analyte	Matrix	Linear range (μg kg^−1^)	R^2^	CCα (μg kg^−1^)	CCβ (μg kg^−1^)
Clenbuterol	Muscle	0.05–1.00	0.9992	0.11	0.13
	Liver	0.10–1.00	0.9987	0.12	0.15
	Kidney	0.05–1.00	0.9985	0.11	0.14
Salbutamol	Muscle	0.25–2.00	0.9990	0.53	0.58
	Liver	0.25–2.00	0.9982	0.55	0.62
	Kidney	0.25–2.00	0.9980	0.54	0.60
Terbutaline	Muscle	0.25–2.00	0.9988	0.52	0.57
	Liver	0.25–2.00	0.9981	0.56	0.63
	Kidney	0.25–2.00	0.9980	0.55	0.61

**Table 3:** Accuracy, recovery, repeatability, and reproducibility for clenbuterol, salbutamol, and terbutaline in muscle, liver, and kidney (*n* = 6).

Analyte	Matrix	QC (μg kg^−1^)	Accuracy (%)	Recovery (Mean ± SD, %)	Repeatability (RSD, %)	Reproducibility (RSDR, %)
Clenbuterol	Muscle	0.10	104.6	104.6 ± 2.9	2.8	5.5
		0.15	103.8	103.8 ± 4.4	4.3	6.5
		0.20	97.8	97.8 ± 5.7	5.8	7.5
	Liver	0.25	106.3	106.3 ± 8.8	8.3	12.0
		0.50	109.3	109.3 ± 9.4	8.6	12.5
		0.75	100.9	100.9 ± 11.8	11.7	15.0
	Kidney	0.10	102.0	102.0 ± 6.1	6.0	9.0
		0.15	101.0	101.0 ± 7.1	7.0	10.0
		0.20	99.0	99.0 ± 7.9	8.0	11.5
Salbutamol	Muscle	0.50	101.1	101.1 ± 5.5	5.5	7.0
		0.75	102.0	102.0 ± 4.7	4.6	6.0
		1.00	105.3	105.3 ± 4.2	4.0	5.8
	Liver	0.25	75.7	75.7 ± 6.8	9.0	14.0
		0.50	75.3	75.3 ± 6.4	8.5	13.5
		0.75	93.5	93.5 ± 9.4	10.0	15.0
	Kidney	0.50	98.0	98.0 ± 7.3	7.5	10.5
		0.75	101.0	101.0 ± 7.1	7.0	10.0
		1.00	103.0	103.0 ± 8.8	8.5	11.5
Terbutaline	Muscle	0.50	67.4	67.4 ± 4.1	6.1	8.0
		0.75	70.3	70.3 ± 3.9	5.5	7.5
		1.00	70.9	70.9 ± 3.3	4.6	7.0
	Liver	0.25	98.7	98.7 ± 7.4	7.5	13.0
		0.50	105.7	105.7 ± 8.5	8.0	13.5
		0.75	103.5	103.5 ± 7.8	7.5	13.0
	Kidney	0.50	96.0	96.0 ± 6.7	7.0	10.5
		0.75	99.0	99.0 ± 7.4	7.5	11.0
		1.00	101.0	101.0 ± 8.1	8.0	11.5

### 2.7 Statistical analysis

Growth-performance data were analyzed using the pen as the experimental unit. Inferential testing was deliberately restricted to Day 20, which was the only weighing carried out before serial euthanasia began and therefore the only time point at which all 24 birds per group, in four replicate pens of six birds, were still present. At Day 20, body weight was compared among groups by one-way analysis of variance (ANOVA) and, where the overall F test was significant (*p* < 0.05), treatment means were separated using Tukey’s honestly significant difference (HSD) *post hoc* test. From Day 22 onwards the number of birds per group fell progressively as animals were removed for scheduled tissue collection, reaching three birds per group at Day 40. A group of this size provides negligible statistical power and an unstable variance estimate, so no ANOVA and no *post hoc* comparison were applied to the later weighings; those body weights are reported descriptively as mean ± SD only. For the same reason, and because birds were removed rather than followed longitudinally so that the data structure was unbalanced across time, no repeated-measures or mixed-effects model was fitted. Prior to analysis, the Day 20 growth-performance data were assessed for normality using the Shapiro–Wilk test and for homogeneity of variances using Levene’s test. No inferential testing was applied to the residue data, which are reported descriptively.

At each sampling point, three birds per group were sampled and their tissues analyzed individually (*n* = 3 birds per group per time point). For each tissue, compound, dose, and sampling day, the three individual results were averaged at the point of reporting and only that group mean was transferred to the archived study file. The underlying per-bird concentrations remained in the instrument data-processing worksheets, were not archived alongside the study record, and could not subsequently be recovered. This is a record-keeping deficiency rather than an analytical one, but its consequence is substantive: because the individual values are unavailable, no measure of dispersion can be calculated for the residue data, and the biological variation in residue concentration between birds within a treatment group cannot be assessed at any sampling point. Residue concentrations are therefore reported throughout as group means of three individually analyzed birds, without standard deviations, and every comparison between compounds, doses, and tissues is descriptive. Concentrations were quantified against matrix-matched calibration curves using stable-isotope-labeled internal standards; results were therefore corrected for matrix effects and analytical recovery through internal-standard normalization and were not additionally adjusted for recovery. Concentrations below the matrix-specific decision limit (CCα) were reported as < CCα and were excluded from quantitative analysis. To compare residue depletion among the three compounds, the terminal decline phase of the group mean concentrations in liver and kidney was fitted by log-linear (first-order) regression of ln-transformed concentration against time, yielding apparent depletion rate constants (k) and apparent depletion half-lives (t½ = ln2/k). Regression was applied only where the group mean concentrations declined monotonically throughout the terminal phase, which was the case in liver and kidney for all six compound–dose combinations. Muscle was excluded because several compound–dose combinations showed non-monotonic changes between consecutive sampling days, and fitting a first-order model to a profile that does not decline monotonically would yield a rate constant without physical meaning. Throughout this paper, k and t½ are reported as apparent descriptive indicators of the rate of residue decline and not as pharmacokinetic parameters, because they derive from destructive sampling of different birds at each time point rather than from serial sampling of the same animals. All statistical analyzes were performed using IBM SPSS Statistics version 29.0 (IBM Corp., Armonk, NY, USA). Growth-performance results are reported as mean ± SD unless otherwise stated.

### 2.8 Ethical approval

All procedures complied with national and institutional animal-welfare guidelines and were approved by the Animal Ethics Committee of the Faculty of Veterinary Medicine, Ss. Cyril and Methodius University in Skopje (Approval No. 6.4/2026). Euthanasia was performed by trained personnel using cervical dislocation in accordance with the AVMA Guidelines for the Euthanasia of Animals (33). Handling before euthanasia was conducted to minimize stress.

## 3 Results

### 3.1 Body-weight performance

Body weights from Day 20 to Day 40 are summarized in Table 4 and Figure 1. At the first weighing (Day 20), before any birds had been removed for residue analysis, all β-agonist-treated groups exhibited lower mean body weights than the untreated control group. This pattern persisted throughout the study, with the control group maintaining the highest mean body weight at each subsequent measurement. None of the tested β-agonists, at either concentration, increased body weight relative to the control group. The magnitude of the differences between treated and control groups varied across sampling times. At several intermediate time points, differences between individual treatment groups and the control group were small relative to the observed within-group variability. Likewise, differences between the 2.5 mg/L and 5 mg/L concentrations of each β-agonist were inconsistent and did not demonstrate a clear concentration-dependent pattern. At Day 20, treatment had a significant effect on body weight (one-way ANOVA, *p* < 0.05), and all β-agonist-treated groups were significantly lighter than the untreated control group. The number of birds contributing to body-weight measurements decreased progressively throughout the study because three birds per treatment group were removed at each scheduled residue-sampling time point. Accordingly, the number of birds per group was 24 on Day 20, 21 on Day 22, 15 on Day 24, 12 on Day 26, 6 on Day 33, and 3 on Day 40. Consequently, body-weight data from later sampling times are presented descriptively as mean ± SD, no ANOVA or *post hoc* comparison having been applied to them, and their interpretation should be approached with caution given the reduced sample size. The increased variability observed in some treatment groups at later ages likely reflects this progressive reduction in bird numbers resulting from serial euthanasia for residue analysis.

**Table 4:** Growth performance (mean ± SD, g) of Ross 308 broiler chickens administered salbutamol, clenbuterol, or terbutaline via drinking water at concentrations of 2.5 or 5 mg/L.

Group	Day 20	Day 22	Day 24	Day 26	Day 33	Day 40
Control (*n* = 24)	952 ± 51	1,088 ± 52	1,462 ± 91	1,700 ± 73	2,727 ± 176	3,610 ± 277
Salbutamol 2.5 mg/L	896 ± 57	979 ± 55	1,368 ± 115	1,547 ± 108	2,446 ± 159	3,237 ± 260
Salbutamol 5 mg/L	880 ± 63	995 ± 71	1,457 ± 97	1,610 ± 112	2,554 ± 215	3,384 ± 326
Clenbuterol 2.5 mg/L	869 ± 52	1,002 ± 80	1,369 ± 101	1,527 ± 145	2,380 ± 476	3,106 ± 626
Clenbuterol 5 mg/L	855 ± 72	982 ± 89	1,367 ± 116	1,560 ± 119	2,449 ± 256	3,214 ± 501
Terbutaline 2.5 mg/L	889 ± 67	1,002 ± 55	1,406 ± 113	1,565 ± 163	2,553 ± 255	3,409 ± 251
Terbutaline 5 mg/L	879 ± 63	1,004 ± 72	1,395 ± 132	1,590 ± 129	2,467 ± 235	3,223 ± 482

Values are presented as group means ± standard deviation (SD). The number of birds contributing to each mean decreased progressively during the study because birds were removed at scheduled intervals for residue-depletion analysis.

Values are expressed as mean ± SD. At Day 20, before initiation of serial euthanasia for residue analysis, all β-agonist-treated groups exhibited significantly lower body weights than the untreated control group (one-way ANOVA followed by Tukey’s HSD test, *p* < 0.05). At subsequent time points, body-weight data are presented descriptively because the number of birds per group decreased due to scheduled tissue sampling. Therefore, no *post hoc* comparisons were performed for the later measurements.

**Figure 1:**
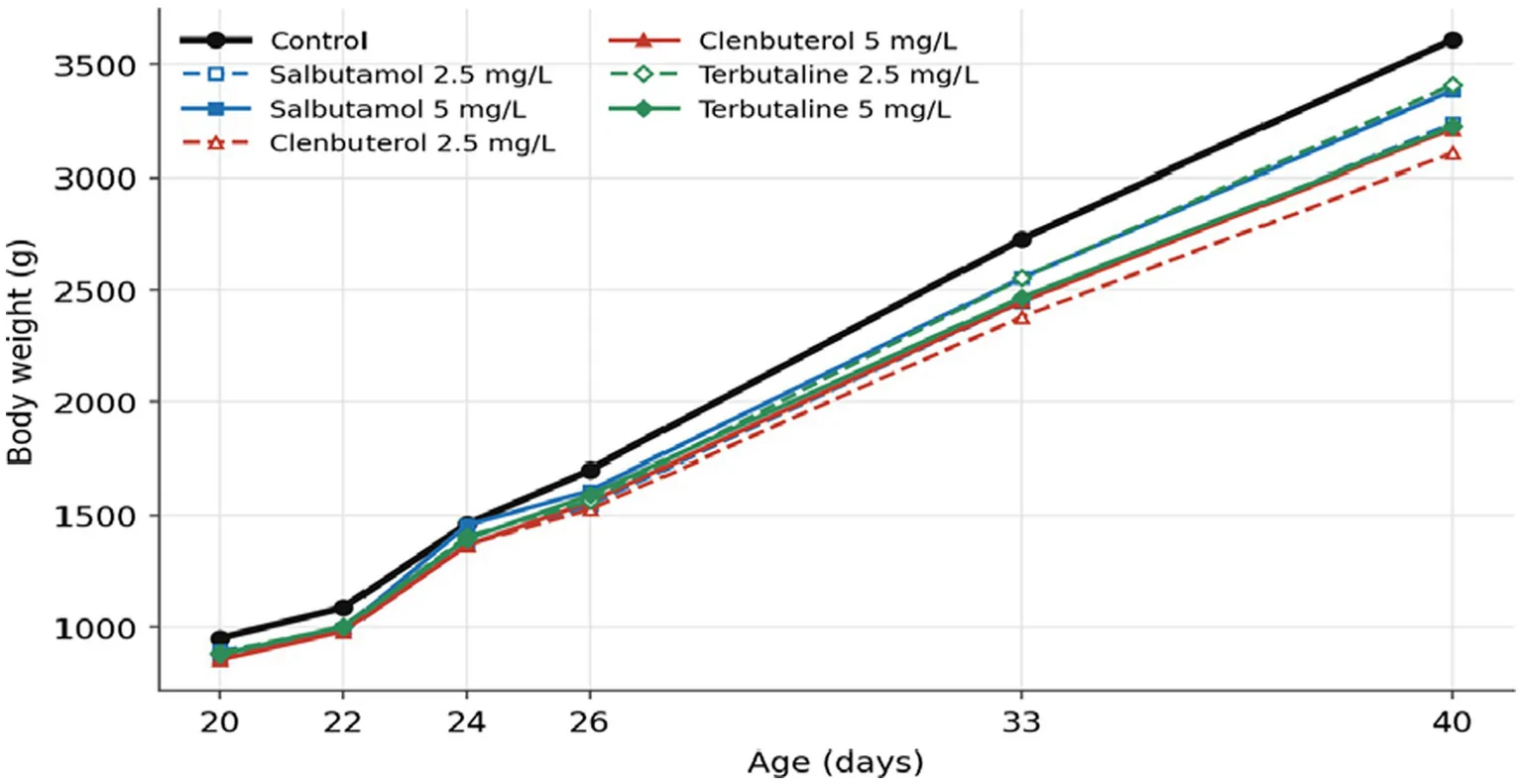
Growth performance (mean body weight, g) of Ross 308 broilers given salbutamol, clenbuterol, or terbutaline at 2.5 or 5 mg/L in the drinking water (Days 13–19), compared with the untreated control, from Day 20 to Day 40. The control exceeded all treated groups at all ages; standard deviations are shown in Table 4.

### 3.2 Method validation

Validation results are summarized in Tables 2, 3, including linearity, coefficient of determination (R^2^), decision limit (CCα), detection capability (CCβ), accuracy, recovery, repeatability, and reproducibility. The method demonstrated excellent linearity for all analyte-matrix combinations, with coefficients of determination (R^2^) of 0.998 or greater. Recovery, accuracy, and precision values were generally within the performance criteria set out in Commission Implementing Regulation (EU) 2021/808. Lower recoveries were observed for terbutaline in muscle and salbutamol in liver; however, these analyte-matrix combinations still exhibited acceptable repeatability and reproducibility, supporting their suitability for confirmatory residue analysis. Method specificity was confirmed in all three matrices. No interfering peaks were detected at the retention times of the target analytes in blank tissue extracts, whereas well-resolved analyte peaks were observed in fortified samples (Supplementary Figure S1). The matrix-specific CCα values presented in Table 2 were used as the decision limits for interpretation and reporting of residue data. Accordingly, concentrations below the corresponding CCα values were reported as non-confirmatory within the regulatory framework rather than as evidence of the complete absence of residues.

### 3.3 Residue depletion

Detectable residues of all three β-adrenergic agonists were observed in edible tissues at the first sampling point following the withdrawal period (Day 21). At both treatment concentrations, residue concentrations declined over the withdrawal period in all three tissues; the decline was consistent and progressive in liver and kidney, whereas breast muscle showed more variable concentrations across the early sampling points. By the final sampling points (Days 39 and 46), concentrations of all analytes in all tissues were below their respective matrix-specific CCα values. Overall, terbutaline exhibited the most rapid depletion, whereas clenbuterol persisted longer in tissues during the withdrawal period.

#### 3.3.1 Kidney

Among the tissues examined, the kidney generally exhibited the highest residue concentrations at the earliest sampling point (Table 5; Figure 2). Detectable concentrations of clenbuterol, salbutamol, and terbutaline were present on Day 21 at both treatment concentrations. Residue concentrations decreased progressively throughout the withdrawal period, with marked reductions observed between Days 23 and 28. Residues of all three analytes were below their respective CCα values by Day 39 and remained below these decision limits until the end of the study. Clenbuterol residues remained detectable for a longer period than those of salbutamol and terbutaline during the early stages of withdrawal.

**Table 5:** Residue concentrations of β‑adrenergic agonists in kidney tissue (μg kg⁻¹) during the withdrawal period following administration via drinking water.

Compound	Dose	Day 21	Day 22	Day 23	Day 25	Day 28	Day 30	Day 39	Day 46
Clenbuterol	2.5 mg/L	117.76	30.59	27.56	2.54	0.13	<0.127	<0.127	<0.127
Clenbuterol	5 mg/L	114.29	30.00	26.18	2.44	<0.127	<0.127	<0.127	<0.127
Salbutamol	2.5 mg/L	110.90	54.06	38.91	10.19	1.66	0.52	<0.48	<0.48
Salbutamol	5 mg/L	94.07	85.89	16.79	9.52	1.88	0.54	<0.48	<0.48
Terbutaline	2.5 mg/L	40.57	26.13	11.10	4.15	0.77	0.24	<0.47	<0.47
Terbutaline	5 mg/L	132.79	44.57	33.26	4.58	<0.47	<0.47	<0.47	<0.47

Values represent group mean concentrations of three birds per treatment group analyzed individually at each sampling time (*n* = 3); individual per-bird values were not retained, so standard deviations are not reported. Results below the corresponding compound-specific CCα values are reported as < CCα (CCα values are presented in Table 2). Because the individual analytical results were not retained, measures of variability could not be calculated and biological variation between birds could not be assessed; the residue data are therefore presented descriptively.

**Figure 2:**
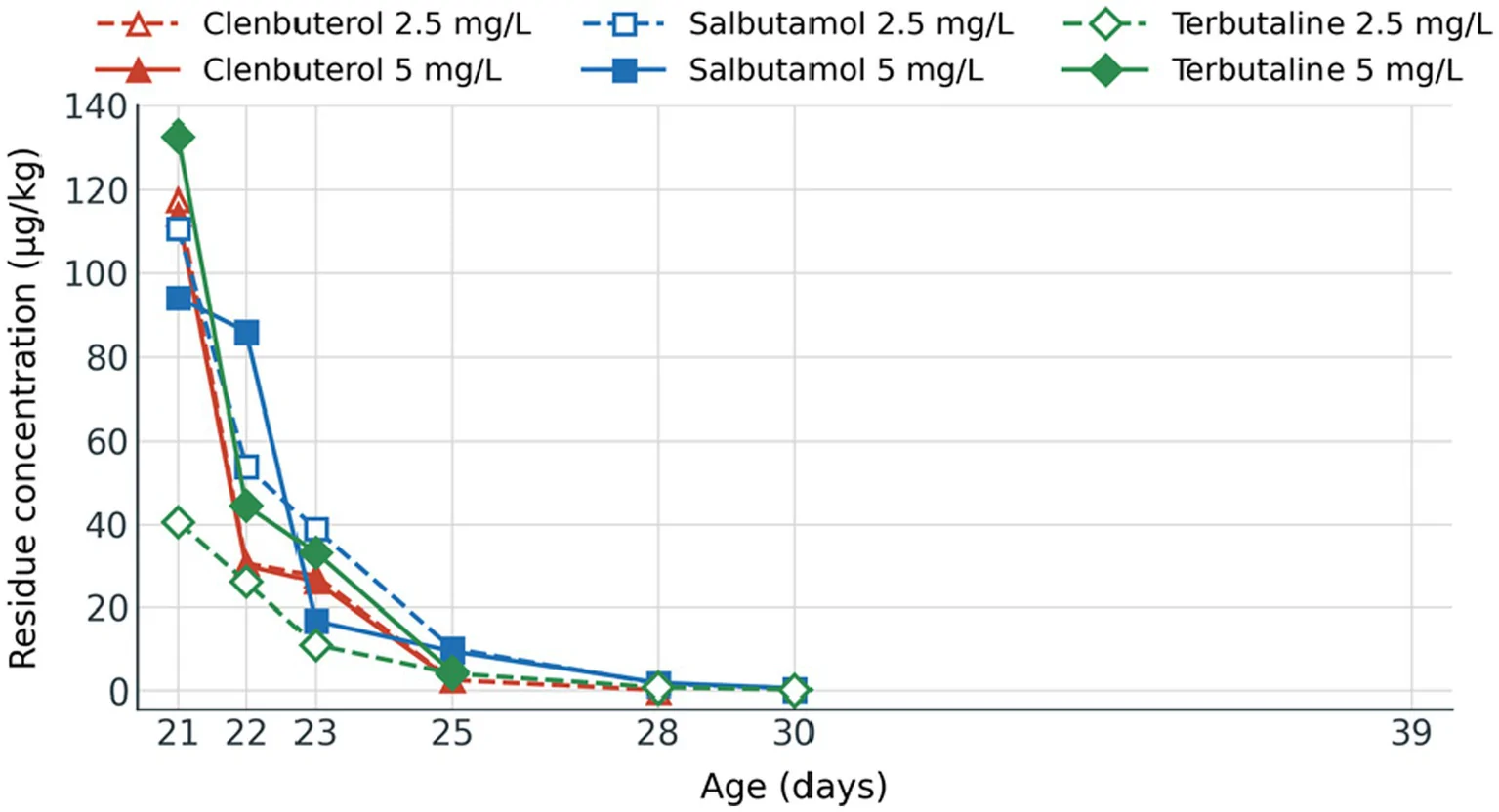
Residue-depletion profiles of clenbuterol, salbutamol, and terbutaline in kidney tissue (μg/kg) following a 7-day administration period (Days 13–19) at concentrations of 2.5 and 5 mg/L in drinking water. Measurements were obtained during the withdrawal period (Days 21–46). Data points below the respective compound-specific CCα values were omitted from the figure for clarity. The *x*-axis is truncated at Day 39 because no analyte exceeded its compound-specific CCα at Day 39 or Day 46. Exact residue concentrations are provided in Table 5.

#### 3.3.2 Liver

Liver residues exhibited a clear time-dependent depletion pattern (Table 6; Figure 3). Residue concentrations of all three β-agonists were highest during the early withdrawal period (Days 21–23) and declined progressively thereafter. A marked reduction in concentrations was observed between Days 25 and 28, and all analytes were no longer present at concentrations exceeding their respective decision limits (CCα) by Days 39 and 46. Compared with breast muscle, residues generally remained detectable longer in the liver, whereas persistence was lower than in the kidney.

**Table 6:** Residue concentrations of β‑adrenergic agonists in liver tissue (μg kg⁻¹) during the withdrawal period following administration via drinking water.

Compound	Dose	Day 21	Day 22	Day 23	Day 25	Day 28	Day 30	Day 39	Day 46
Clenbuterol	2.5 mg/L	120	85	65	28	5.5	2.0	<0.133	<0.133
Clenbuterol	5 mg/L	160	110	85	38	7.5	3.0	<0.133	<0.133
Salbutamol	2.5 mg/L	150	105	78	34	6.5	1.8	<0.45	<0.45
Salbutamol	5 mg/L	190	135	98	42	8.0	2.4	<0.45	<0.45
Terbutaline	2.5 mg/L	90	62	47	17	1.4	0.5	<0.45	<0.45
Terbutaline	5 mg/L	120	82	63	22	2.0	0.8	<0.45	<0.45

Values represent group mean concentrations of three birds per treatment group analyzed individually at each sampling time (*n* = 3); individual per-bird values were not retained, so standard deviations are not reported. Results below the corresponding compound-specific CCα values are reported as < CCα. Because the individual analytical results were not retained, measures of variability could not be calculated and biological variation between birds could not be assessed; the residue data are therefore presented descriptively.

**Figure 3:**
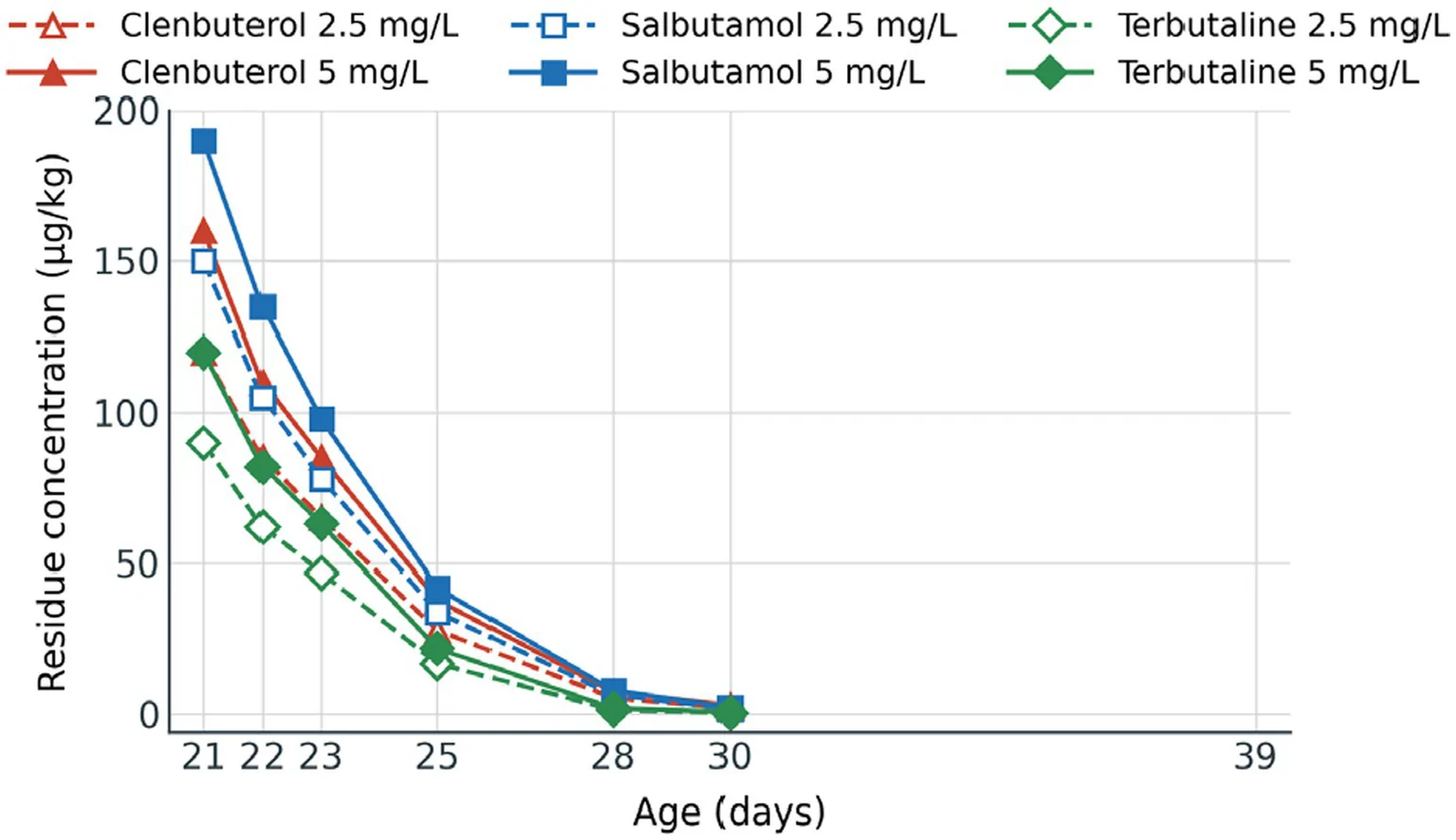
Liver residue concentrations (μg/kg) of clenbuterol, salbutamol, and terbutaline during the withdrawal period (Days 21–46) following administration in drinking water at 2.5 and 5 mg/L. Values below the compound-specific CCα are not displayed. The x-axis is truncated at Day 39 because no analyte exceeded its compound-specific CCα at Day 39 or Day 46. Exact values are provided in Table 6.

#### 3.3.3 Breast muscle

Detectable residues of all three β-agonists were present in breast muscle during the early withdrawal period (Table 7; Figure 4). Muscle concentrations were more variable across consecutive sampling days than those observed in liver and kidney and did not follow a strictly progressive decline. Most analytes fell below their respective compound-specific CCα values by Day 30; clenbuterol administered at the higher concentration (5 mg/L) remained detectable at Day 30 (2.0 μg/kg) before declining no longer present at concentrations exceeding the decision limit (CCα) by Day 39. All analytes were below CCα for the remainder of the study (Days 39 and 46). With the exception of the high-dose clenbuterol profile, residues generally persisted for a shorter period in breast muscle than in liver and kidney.

**Table 7:** Residue concentrations of β‑adrenergic agonists in breast muscle (μg kg⁻¹) during the withdrawal period following administration via drinking water.

Compound	Dose	Day 21	Day 22	Day 23	Day 25	Day 28	Day 30	Day 39	Day 46
Clenbuterol	2.5 mg/L	30.0	16.81	26.90	5.0	0.55	<0.07	<0.07	<0.07
Clenbuterol	5 mg/L	25.0	11.54	32.91	20.0	17.83	2.0	<0.07	<0.07
Salbutamol	2.5 mg/L	45.0	40.22	23.66	8.0	1.16	<0.47	<0.47	<0.47
Salbutamol	5 mg/L	70.0	66.08	12.29	6.0	1.56	<0.47	<0.47	<0.47
Terbutaline	2.5 mg/L	18.0	14.93	21.22	6.0	0.88	<0.46	<0.46	<0.46
Terbutaline	5 mg/L	25.0	15.59	36.77	7.0	<0.46	<0.46	<0.46	<0.46

Values represent group mean concentrations of three birds per treatment group analyzed individually at each sampling time (*n* = 3); individual per-bird values were not retained, so standard deviations are not reported. Results below the corresponding compound-specific CCα values are reported as < CCα. Because the individual analytical results were not retained, measures of variability could not be calculated and biological variation between birds could not be assessed; the residue data are therefore presented descriptively.

**Figure 4:**
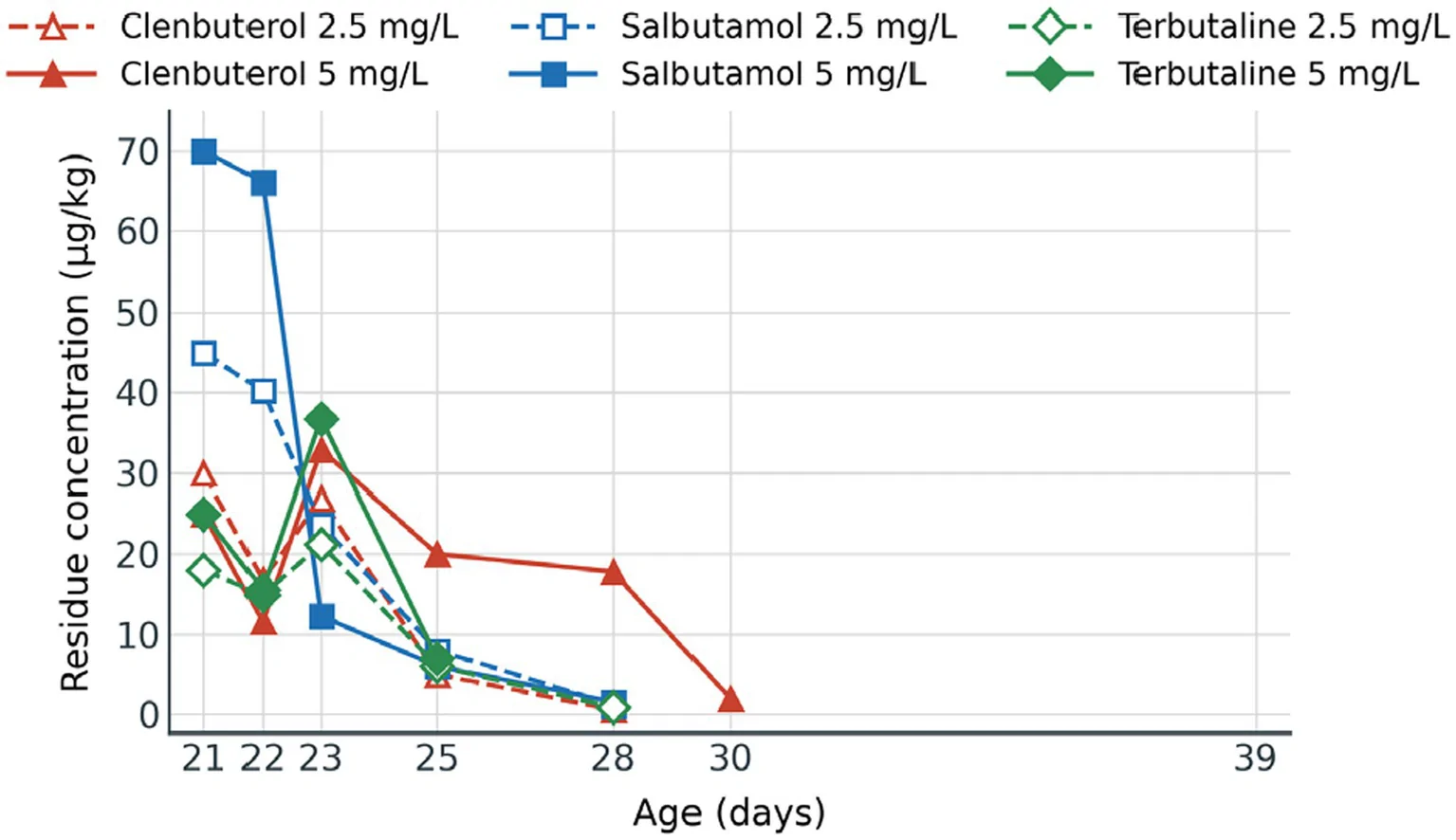
Residue-depletion profiles of clenbuterol, salbutamol, and terbutaline in breast muscle (μg/kg) following administration via drinking water at concentrations of 2.5 and 5 mg/L. Residue concentrations were determined during the withdrawal period (Days 21–46). Data points below the respective compound-specific CCα values are not shown. The *x*-axis is truncated at Day 39 because no analyte exceeded its compound-specific CCα at Day 39 or Day 46. Exact residue concentrations are presented in Table 7.

#### 3.3.4 Chromatographic confirmation

All three β-adrenergic agonists produced sharp and symmetrical MRM peaks at their characteristic retention times, with no significant matrix interference observed in blank tissue extracts (Supplementary Figure S1). Peak intensities were greatest during the early withdrawal period (Days 21–23) and decreased progressively at subsequent sampling times. By Days 39 and 46, no signals exceeding the corresponding compound-specific CCα values were detected in any of the examined tissues. Representative confirmation chromatograms for liver, breast muscle, and kidney are presented in Supplementary Figures S2–S4.

#### 3.3.5 Apparent depletion kinetics

Log-linear regression of the group-mean residue concentrations during the terminal decline phase yielded apparent depletion rate constants and apparent half-lives for liver and kidney (Table 8). Fits were strong for both tissues (R^2^ = 0.958–0.998). In liver, apparent half-lives were shortest for terbutaline (1.14–1.18 days) and longest for clenbuterol (1.50–1.54 days), with salbutamol intermediate (1.41–1.42 days), consistent with the descriptive observation that terbutaline depleted most rapidly. In the kidney, apparent half-lives were shorter overall (0.72–1.22 days), and the rank order differed, with clenbuterol showing the fastest apparent kidney depletion, indicating that relative persistence varied between organs. These parameters are apparent descriptive indicators of the rate of residue decline, derived from group-mean concentrations (*n* = 3 birds per time point) rather than from plasma or individual-animal data, and should be read as comparators between compounds and tissues rather than as formal pharmacokinetic estimates. Muscle was excluded from the regression analysis. Several compound–dose combinations showed non-monotonic concentration changes between consecutive sampling days (Table 7), and a first-order model fitted to a profile that does not decline monotonically would return a rate constant with no physical meaning. No log-linear model was therefore fitted to any muscle dataset, and the muscle results are described qualitatively only.

**Table 8:** Apparent depletion rate constants (k) and apparent depletion half-lives (t½) for clenbuterol, salbutamol, and terbutaline in liver and kidney, obtained by log-linear regression of group mean residue concentrations during the terminal decline phase.

Compound	Matrix	Dose (mg/L)	k (day^−1^)	Apparent t½ (days)	R^2^
Clenbuterol	Liver	2.5	0.462	1.50	0.992
Clenbuterol	Liver	5	0.450	1.54	0.992
Salbutamol	Liver	2.5	0.491	1.41	0.988
Salbutamol	Liver	5	0.488	1.42	0.990
Terbutaline	Liver	2.5	0.605	1.14	0.982
Terbutaline	Liver	5	0.587	1.18	0.984
Clenbuterol	Kidney	2.5	0.961	0.72	0.987
Clenbuterol	Kidney	5	0.917	0.76	0.958
Salbutamol	Kidney	2.5	0.596	1.16	0.998
Salbutamol	Kidney	5	0.573	1.21	0.973
Terbutaline	Kidney	2.5	0.570	1.22	0.998
Terbutaline	Kidney	5	0.812	0.85	0.976

Muscle was not modeled owing to irregular concentration profiles. Values are apparent descriptive indicators of the rate of residue decline, derived from group-mean concentrations (*n* = 3 birds per time point); they are not pharmacokinetic parameters.

## 4 Discussion

The present study evaluated the growth-performance effects, tissue distribution, and residue-depletion characteristics of three β-adrenergic agonists, salbutamol, clenbuterol, and terbutaline, in Ross 308 broiler chickens following administration in drinking water for seven consecutive days. Under the conditions examined, none of the compounds improved body weight relative to the untreated control group, and no consistent concentration-dependent response was observed. In contrast, clear differences were evident in tissue residue profiles and depletion behavior among the three compounds. The absence of a growth-promoting effect is consistent with previous reports indicating that poultry respond less predictably to β-agonist administration than mammalian livestock species. In cattle, pigs, and sheep, β₂-adrenergic agonists can enhance lean tissue accretion by stimulating protein synthesis and reducing protein degradation, thereby improving growth performance and carcass composition (1, 6–10). In broilers, however, the distribution and responsiveness of β-adrenergic receptors appear to differ from those reported in mammals, and published studies have yielded inconsistent results regarding performance enhancement (1, 11). The lack of a clear dose–response relationship in the present study further suggests that increasing exposure within the tested range did not produce measurable improvements in body weight gain. These findings are consistent with previous investigations involving salbutamol, clenbuterol, and terbutaline in poultry, in which metabolic or compositional effects were often more evident than changes in live weight (6, 11, 29, 37). A further explanation specific to the birds used here concerns the genetic background of the strain. Ross 308 is a modern commercial broiler that has been subject to decades of intensive selection for growth rate, breast-muscle yield, and feed efficiency (38). Selection of this intensity is likely to have moved protein accretion close to the physiological maximum that nutrient supply, cardiovascular capacity, and skeletal frame will support, so that the anabolic axis on which β₂-agonists act is already operating near its upper limit. Under such a ceiling effect, a nutrient-repartitioning stimulus has little residual capacity left to exploit, and the complete absence of a weight response in the treated groups is consistent with that interpretation. The same reasoning predicts a smaller response in a genetically optimized modern strain than in the slower-growing birds used in the earlier poultry studies of β-agonists (6), and it is compatible with the observation that, where effects have been reported in poultry, they have more often been compositional or metabolic than reflected in live weight. Distinct tissue-specific residue patterns were observed for all three compounds. Residue concentrations were generally highest in kidney and liver during the early withdrawal period, whereas breast muscle consistently contained the lowest concentrations and showed the shortest persistence. Although tissue-residue measurements do not permit direct assessment of absorption, distribution, metabolism, or excretion processes, the observed distribution pattern is broadly consistent with previous reports describing greater accumulation and persistence of β-agonists in highly perfused organs such as the liver and kidney than in skeletal muscle (29–31). These differences likely reflect a combination of tissue perfusion, cellular uptake, tissue binding, metabolic activity, and excretory processes rather than any single underlying mechanism. An important observation was that residue concentrations did not always increase in proportion to the nominal drinking-water concentration. In several instances, birds receiving 5 mg/L exhibited residue concentrations similar to or lower than those observed in birds receiving 2.5 mg/L. Because administration occurred through shared drinking water and individual water consumption was not measured, actual exposure likely varied among birds. Consequently, nominal treatment concentration should not be regarded as a direct indicator of absorbed dose. Variability in individual water intake may therefore have contributed to the inconsistent concentration-response relationships observed between treatment groups and should be considered when interpreting the residue data. Among the compounds examined, clenbuterol generally exhibited the greatest persistence during the withdrawal period, whereas terbutaline depleted most rapidly. This finding is consistent with previous studies that reported prolonged retention of clenbuterol compared with other β-agonists in edible tissues (29, 30). The greater persistence of clenbuterol has been attributed to physicochemical characteristics such as higher lipophilicity and stronger tissue binding compared with more polar compounds including salbutamol and terbutaline (30, 39). Although these mechanisms were not investigated directly in the present study, they provide a plausible explanation for the observed differences in residue persistence among compounds. From a residue-monitoring perspective, the longer persistence of clenbuterol further underscores its continued importance as a target analyte in surveillance programs aimed at detecting the illegal use of β-agonists in food-producing animals. Residues of all three compounds declined progressively throughout the withdrawal period and were below the corresponding matrix-specific decision limits (CCα) by the end of the study. To provide a quantitative comparison of depletion behavior, the terminal decline phase of liver and kidney residues was described using log-linear regression. The resulting apparent depletion half-lives (Table 8) supported the descriptive observations, indicating more rapid depletion of terbutaline and greater persistence of clenbuterol. However, these values should be interpreted with caution because they were derived from group-mean tissue concentrations rather than from individual-animal concentration data or serial pharmacokinetic sampling. Consequently, the calculated half-lives are descriptive indicators of relative depletion behavior and should not be interpreted as formal pharmacokinetic parameters. The use of CCα as the principal reporting threshold provides a regulatory framework for interpreting analytical results. Under Commission Implementing Regulation (EU) 2021/808, CCα represents the decision limit above which a sample may be classified as non-compliant with a defined statistical certainty (40). Therefore, residue concentrations reported as below CCα should not be interpreted as proof of complete absence of the analyte but rather as concentrations below the threshold required for confirmatory regulatory action. This distinction is also the reason why CCα, rather than a limit of detection or a limit of quantification, is used as the reporting threshold throughout the present study. Clenbuterol, salbutamol, and terbutaline are prohibited substances for which no maximum residue limit is established, so the analytical outcome is expressed against the decision limit rather than against an instrumental threshold. LOD and LOQ describe what the instrument is able to detect and to quantify reliably; CCα defines the concentration above which a laboratory may declare a sample non-compliant with a stated probability of a false non-compliant result. The two are not interchangeable. A result reported here as less than CCα therefore means only that the residue was below the threshold at which regulatory action could be taken; it does not indicate that no residue was present, and it is not equivalent to a result below the limit of detection. This distinction is important when evaluating residue persistence and interpreting surveillance data. Several limitations should be considered when interpreting the findings. First, administration through shared drinking water prevented determination of individual intake. Water consumption can be influenced by body weight, social hierarchy, and environmental conditions, making group-level exposure only an approximation of individual dose. Second, residue measurements were based on a limited number of birds at each sampling point (*n* = 3). Because only group-mean concentrations were retained and individual residue values were not preserved, measures of variability, such as standard deviations, could not be calculated. Consequently, biological variation in residue concentration between individual birds could not be assessed at any sampling point. This is the principal limitation of the residue dataset. The depletion profiles describe the central tendency of each group but carry no information about the spread around it, so the apparent differences between compounds, doses, and tissues cannot be supported by statistical testing and must be read as descriptive rather than as definitive estimates of residue kinetics. Recovery of the individual analytical results was attempted but was not possible, and future work of this kind should archive per-bird concentrations so that within-group variability can be quantified. Third, the study employed a mixed-sex bird population and did not evaluate sex-specific responses, despite known differences in growth performance between male and female broilers. Fourth, only a single seven-day exposure regimen was investigated, and longer treatment periods could result in different tissue-loading and depletion patterns. Finally, because only tissue residues were measured and no plasma concentration data were collected, the study should not be regarded as a formal pharmacokinetic investigation and does not provide a basis for statistically derived withdrawal-period determination. Instead, it provides comparative residue-depletion information relevant to residue surveillance, regulatory monitoring programs, and food-safety risk assessment. Overall, the findings demonstrate that salbutamol, clenbuterol, and terbutaline differ substantially in their tissue distribution and persistence in broiler chickens despite producing no measurable growth-promoting effect under the conditions evaluated. The comparative residue-depletion data generated in this study contribute to the understanding of β-agonist behavior in poultry tissues and may assist regulatory authorities in developing risk-based monitoring strategies for the detection of illicit β-agonist use in poultry production systems (22–24, 40).

## 5 Conclusion

Under the conditions of the present study, salbutamol, clenbuterol, and terbutaline administered via drinking water at nominal concentrations of 2.5 and 5 mg/L did not enhance growth performance in Ross 308 broiler chickens. This finding is consistent with previous studies reporting limited and inconsistent growth-promoting responses to β-adrenergic agonists in poultry (29, 37) and may reflect the reduced responsiveness of avian β-adrenergic systems compared with those of mammalian livestock species (1, 11). The growth-performance findings should nevertheless be interpreted with caution due to the declining sample size over time and the mixed-sex study population. Residue analysis using a validated LC–MS/MS method detected salbutamol, clenbuterol, and terbutaline residues in kidney, liver, and breast muscle during the withdrawal period. Residue concentrations declined over the withdrawal period in all examined tissues, although breast-muscle profiles were more variable across consecutive sampling days than those in liver and kidney. Kidney and liver accumulated substantially higher residue concentrations than breast muscle during the early withdrawal period, although apparent depletion kinetics indicated that persistence varied according to both tissue and compound; terbutaline depleted most rapidly, whereas clenbuterol showed the greatest persistence, and for clenbuterol the apparent half-life in liver (1.50–1.54 days) exceeded that in kidney (0.72–0.76 days). These findings are consistent with previous reports describing tissue-dependent accumulation and persistence of β-agonists in food-producing animals (29–31). Unexpectedly, several residue concentrations did not increase proportionally with the nominal drinking-water concentration, and in some tissues the 5 mg/L groups showed lower residues than the 2.5 mg/L groups. Because the compounds were administered through shared drinking water and individual water intake was not measured, the actual exposure of individual birds likely varied, so that nominal treatment concentration may not have accurately reflected the dose received; comparable variability has been reported in group-administered exposure studies in which individual consumption could not be controlled. By Day 46, residue concentrations of all three compounds were no longer present at concentrations exceeding their respective matrix-specific decision limits (CCα) in all tissues examined. The present study generates comparative residue-depletion profiles for three β-adrenergic agonists under standardized exposure conditions and contributes to the understanding of residue persistence in edible poultry tissues. These findings are relevant to residue-monitoring programs, regulatory surveillance, and food-safety risk assessment of β-agonist residues in poultry-derived food products (22–24, 40). Because the study was based exclusively on tissue residue measurements and did not include plasma concentration data, and residue depletion was characterized only by apparent log-linear modeling of group mean concentrations, the results should not be interpreted as a formal pharmacokinetic evaluation or as a basis for statistically derived withdrawal-period determination. Rather, they provide descriptive information on tissue residue depletion following short-term exposure in broiler chickens. Future studies incorporating plasma concentration measurements, larger sample sizes, and formal depletion modeling would provide a stronger basis for characterizing residue kinetics and establishing scientifically supported withdrawal periods.

## Data Availability

Data supporting the findings of this study are available from the corresponding author upon reasonable request. Requests to access the datasets should be directed to zhajrulai@fvm.ukim.edu.mk.
