# Supplementary material for: Comparative residue depletion and tissue distribution of three β-adrenergic agonists in broiler chickens
Source: Front Vet Sci. 2026 Sep 23;13:1967907. doi: 10.3389/fvets.2026.1967907 (PMC13643264; doi:10.3389/fvets.2026.1967907)
Supplement: Supplementary file 1 [file Supplementary_file_1.docx]

**Supplementary Material**

**Figure S1.** Specificity of the validated LC–MS/MS method for clenbuterol, salbutamol, and terbutaline in (a) breast muscle, (b) liver, and (c) kidney. For each analyte, representative multiple reaction monitoring (MRM) chromatograms are shown for a blank tissue extract (left) and a tissue sample fortified at the validated fortification level (right). Each panel displays the quantifier (upper trace) and qualifier (lower trace) transitions. No interfering signals were observed at the retention times of the target analytes in any blank matrix, confirming method specificity. Monitored transitions (quantifier/qualifier): clenbuterol m/z 277.0 → 203.0 / 131.9; salbutamol m/z 240.0 → 148.0 / 166.0; terbutaline m/z 226.0 → 152.0 / 107.0.

**(a) Breast muscle**

| **Blank tissue extract** | **Fortified tissue extract** |
| --- | --- |
| ***Clenbuterol*** | |
|  |  |
| ***Salbutamol*** | |
|  |  |
| ***Terbutaline*** | |
|  |  |

**(b) Liver**

| **Blank tissue extract** | **Fortified tissue extract** |
| --- | --- |
| ***Clenbuterol*** | |
|  |  |
| ***Salbutamol*** | |
|  |  |
| ***Terbutaline*** | |
|  |  |

**(c) Kidney**

| **Blank tissue extract** | **Fortified tissue extract** |
| --- | --- |
| ***Clenbuterol*** | |
|  |  |
| ***Salbutamol*** | |
|  |  |
| ***Terbutaline*** | |
|  |  |

**Figure S2.** Representative MRM confirmation chromatograms for the three β-agonists in liver. For each analyte the reference standard is shown together with incurred samples at the low (2.5 mg/L) and high (5 mg/L) dose on selected sampling days. Each panel displays the two acquired MRM transitions (quantifier and qualifier) with the analyte retention time, confirming identity and specificity.

**(a) Clenbuterol — liver**

MRM transitions 276.97 → 202.95 (quantifier) and 276.97 → 131.87 (qualifier); retention time ≈ 2.5 min.

**(b) Salbutamol — liver**

MRM transitions 240.03 → 147.96 (quantifier) and 240.03 → 165.98 (qualifier); retention time ≈ 1.35 min.

**(c) Terbutaline — liver**

MRM transitions 226 → 152 (quantifier) and 226 → 106.97 (qualifier); retention time ≈ 1.33 min.

**Figure S3.** Representative MRM confirmation chromatograms for the three β-agonists in breast muscle, shown for incurred samples at the low (2.5 mg/L) and high (5 mg/L) dose on selected sampling days. Each panel displays the two acquired MRM transitions (quantifier and qualifier) with the analyte retention time; the corresponding reference standards are shown in Figure S2.

**(a) Clenbuterol — breast muscle**

MRM transitions 276.97 → 202.95 (quantifier) and 276.97 → 131.87 (qualifier); retention time ≈ 2.47 min.

**(b) Salbutamol — breast muscle**

MRM transitions 240.03 → 147.96 (quantifier) and 240.03 → 165.98 (qualifier); retention time ≈ 1.35 min.

**(c) Terbutaline — breast muscle**

MRM transitions 226 → 152 (quantifier) and 226 → 106.97 (qualifier); retention time ≈ 1.34 min.

**Figure S4.** Representative MRM confirmation chromatograms for the three β-agonists in kidney, shown for incurred samples at the low (2.5 mg/L) and high (5 mg/L) dose on selected sampling days. Each panel displays the two acquired MRM transitions (quantifier and qualifier) with the analyte retention time; the corresponding reference standards are shown in Figure S2.

**(a) Clenbuterol — kidney**

MRM transitions 276.97 → 202.95 (quantifier) and 276.97 → 131.87 (qualifier); retention time ≈ 2.44 min.

**(b) Salbutamol — kidney**

MRM transitions 240.03 → 147.96 (quantifier) and 240.03 → 165.98 (qualifier); retention time ≈ 1.35 min.

**(c) Terbutaline — kidney**

MRM transitions 226 → 152 (quantifier) and 226 → 106.97 (qualifier); retention time ≈ 1.34 min.
